# Case-mix and outcome variability in people with diabetic foot complications in England and Scotland

**DOI:** 10.1093/eurpub/ckac131.129

**Published:** 2022-10-25

**Authors:** B Meza, C Heiss, M Joy, M Feher, G Leese, S Cunningham, S DeLusignan, F Carinci

**Affiliations:** 1 Primary Care Health Sciences, University of Oxford, Oxford, UK; 2 Surrey and Sussex Healthcare NHS Trust, East Surrey Hospital, Redhill, UK; 3 Division of Population Health and Genomics, University of Dundee, Dundee, UK; 4 Department of Statistical Sciences, University of Bologna, Bologna, Italy

## Abstract

**Background:**

We assessed the difference in survival rates for people with diabetes experiencing LEA in England and Scotland, using large databases held by the Royal College of General Practitioners (RCGP) and the Scottish Diabetes Register (SCI-DC).

**Methods:**

Observational retrospective study of T2D adults 18+ years with LEA between 1/1/2008-1/1/2018 from 1,800 general practices in England (7.4%) and all primary, secondary care units in Scotland. Significance tests were carried out using univariate odds ratios within each database.

**Results:**

On 1/1/2018, N = 127,100 people with T2D were registered alive in RCGP, with N = 1,052 (832 per 100,000) experiencing prior LEA, vs N = 2,200 (783 per 100,000) out of 280,908 in SCI-DC. Among them, England recorded N = 405 patients (72.5%) with prior DFU diagnosis vs N = 993 in Scotland (74.3%), with a median time DFU to LEA of 2.0 vs 2.4 years. The median time spent with LEA was 3.4 years in England vs 3.9 years in Scotland. After including those dying earlier, different univariate patterns were found for England and Scotland. In both networks, increased risk was found for those aged 50+ at first LEA, with prior history of acute myocardial infarction, peripheral arterial disease, ischemic heart disease, cerebrovascular event, higher glomerular filtration rate and major LEA first. In England, reduced risks were found for males (OR = 0.77, 95%CI: 0.64-0.93) and people with retinopathy (0.69; 0.57-0.82), while higher risk were found for hypertension (1.29; 1.09-1.54). In Scotland, lower risks were found for obese (0.59; 0.52-0.66) and those with DFU after LEA (0.74; 0.62-0.89), vs higher rates among those of non-white ethnicity (1.63; 1.19-2.23) and dyalisis (2.31; 1.75-3.07).

**Conclusions:**

Notable differences were found between England and Scotland in terms of characteristics associated with different outcomes following LEA among T2D adults. Multivariate analyses of aggregate patterns are currently ongoing to adjust for potential confounding.

**Key messages:**

• Routine datasets from England and Scotland showed a differential impact of case-mix characteristics on lower extremity amputations among adults with Type 2 diabetes.

• Information available from different clinical networks can be mapped against the available standard sets to compare health care outcomes of people with different complex conditions.

